# VP-SOM: View-Planning Method for Indoor Active Sparse Object Mapping Based on Information Abundance and Observation Continuity

**DOI:** 10.3390/s23239415

**Published:** 2023-11-26

**Authors:** Jiadong Zhang, Wei Wang

**Affiliations:** The Robotics Institute, School of Mechanical Engineering and Automation, Beihang University, Beijing 100190, China; zhangjiadong@buaa.edu.cn

**Keywords:** view planning, object active mapping, planning under uncertainty, sparse object model

## Abstract

Active mapping is an important technique for mobile robots to autonomously explore and recognize indoor environments. View planning, as the core of active mapping, determines the quality of the map and the efficiency of exploration. However, most current view-planning methods focus on low-level geometric information like point clouds and neglect the indoor objects that are important for human–robot interaction. We propose a novel View-Planning method for indoor active Sparse Object Mapping (VP-SOM). VP-SOM takes into account for the first time the properties of object clusters in the coexisting human–robot environment. We categorized the views into global views and local views based on the object cluster, to balance the efficiency of exploration and the mapping accuracy. We developed a new view-evaluation function based on objects’ information abundance and observation continuity, to select the Next-Best View (NBV). Especially for calculating the uncertainty of the sparse object model, we built the object surface occupancy probability map. Our experimental results demonstrated that our view-planning method can explore the indoor environments and build object maps more accurately, efficiently, and robustly.

## 1. Introduction

To better serve humans in coexisting human–robot environments, service robots need to comprehend objects’ semantics and geometric information and to execute human interaction commands, like “bring the cup” or “go to the side of the table”. Traditional maps that only contain geometric landmarks, such as points, lines, and surfaces, cannot meet the needs of human–robot interaction. Thus, the object map containing object models and semantic labels is increasingly vital. Object landmarks in the map enrich the map’s semantic information and enhance the robustness of robot localization. The types of object models in object mapping in previous works can be divided into two categories: dense object models and sparse object models. A dense object model’s surface is finely modeled using elements like surfels or voxels, as seen in Co-fusion [1], MaskFusion [2], etc. Dense object modeling costs massive computing resources and increases the costs of map construction and maintenance. The detailed surface of the object needs further processing, to obtain the object’s pose and size required for robot manipulation. As a result, a dense object model cannot directly serve human–robot interaction. By contrast, sparse object models store only the object’s center, orientation, and size. A sparse object model generally takes the form of a cuboid or an ellipsoid and is constructed by multi-view geometry methods, such as Cubeslam [3] and QuadricSLAM [4,5,6]. Sparse object models can also be calculated directly by the bounding box of an object point cloud obtained by clustering the object’s internal point clouds, such as EAO-SLAM [7], or extracted from SDF models of objects using continuous signed distance functions [8]. Sparse object models require fewer computing resources and can directly serve human–robot interaction.

However, observation views in active mapping highly impact the quality of the sparse object model. Firstly, objects, as individual and complete units, often extend beyond the sensor’s Field of View (FoV). Also, indoor objects frequently overlap and occlude one another. Therefore, a bad observation view can readily lead to incomplete object extraction and incorrect object segmentation. Secondly, the perception view of indoor mobile robots is limited by the robot’s movement trajectory, so the continuous observation of objects is hard to realize, which leads to the issue of unobservability [9], increasing the risks of erroneous data association and the probability of objects being erroneously deleted due to insufficient observations. Continuous multi-angle observation of objects can mitigate the unobservability, improve the accuracy of the object model, and reduce the estimation errors in the size and depth of sparse object models. However, no view-planning method currently targets indoor sparse object models. Therefore, exploring a view-planning method for indoor sparse object models is critically important.

The Next-Best View (NBV) for active mapping is defined as the new view that offers the richest information and the observation most-continuous with previous views. The pose of the NBV is the position and orientation vector where the sensor acquires new data. The classic view-planning method, as the core of active mapping, involves selecting candidate views and calculating the NBV from these candidates. Previous view-planning methods have mostly relied on low-level information, such as the map frontier [10,11] and the grid occupancy probability [12]. Object information has been neglected or has only been used in the form of object surface deficits, which is unsuitable for sparse object models. Notably, such sparse object models only contain size and pose information and cannot be directly integrated into the view-evaluation function. Furthermore, indoor objects are often arranged randomly: thus, they form multiple object clusters in coexisting human–robot environments. The traditional methods do not take into account the characteristics of objects in real indoor scenes. For example, they select candidates around individual objects. Finally, the traditional methods focus only on the endpoints of exploration and neglect each position during movement, leading to the problem of unobservability.

This paper proposes a novel View-Planning method for indoor Sparse Object Mapping (VP-SOM) based on information abundance and observation continuity. VP-SOM aims to solve the view-planning problem of sparse object models in coexisting human–robot scenarios. We first studied the characteristics of objects in coexisting human–robot environments. We propose the concept of the object cluster, to take into account the uses and activity attributes of different objects. NBVs are divided into the Global Best View (GBV), which aims to explore more information, and the Local Best View (LBV), which aims to observe object clusters continuously. We developed a view-evaluation function incorporating the uncertainty of the object model, the observation Line of Sight (LoS), non-occlusion, and the effects of data association. In particular, we built an object surface occupancy probability map, to incorporate sparse object models conducive to human–robot interaction into the view-evaluation function.

In summary, we made the following contributions:

- We propose a view-planning method for indoor object active mapping, including the selection of candidate views and NBVs.
- We propose a view-evaluation function for sparse object models, to ensure the information abundance and observation continuity of objects.
- We validated our method through the accuracy, precision of object maps, and observation efficiency in the simulation environments.

The rest of the paper is organized as follows. Section 2 discusses related work about information-entropy-based methods and object-based methods. Section 3 presents our view-planning method for indoor sparse object mapping, including the view-evaluation function, GBV, LBV, and termination condition. Section 4 and Section 5, respectively, explain the two components of the view-evaluation function. Section 6 constructs the active mapping system based on VP-SOM. Section 7 contains the experimental results between VP-SOM and two other view-planning methods.

## 2. Related Work

### 2.1. Information-Entropy-Based Methods

Information entropy, as proposed by Shannon [13], provides a measure of uncertainty that can be used to evaluate the uncertainty in maps. View-planning methods based on information entropy tend to select views as the NBV that most rapidly reduces map uncertainty. Therefore, information gain, which means the reduction in information entropy, becomes an important indicator in the view-evaluation function for these approaches.

Early work [10,11] used the map frontier and the reachable space of robot motion as candidate views. Based on these methods, Bourgault et al. [14] first introduced the entropy of the map and robot pose into the view-evaluation function. The map entropy is calculated by the occupancy probability of a grid map, and the robot pose entropy is determined from the covariance matrix of robot pose estimates from particle filter-based SLAM.

To characterize pose entropy in a graph-based SLAM system, Carrillo et al. [15] attempted employing the Renyi entropy. Subsequently, Isler et al. [16] extended the entropy-based method into 3D space. They used the occupancy probability of each voxel in OctoMap to calculate their information entropy and considered factors such as the camera FoV and the object occlusion by assigning different weights to different voxels. Wang et al. [17] applied this 3D method to large-scale industrial scenes. Zheng et al. [18] introduced semantic segmentation entropy of the environment, which is measured by the semantic labels of voxels and their corresponding confidence probability in semantic segmentation.

Similar to information-entropy-based methods, the Theory of Optimal Experimental Design (TOED) can also be used to account for the utility of performing the active mapping action, and each action is considered as a stochastic design, while comparisons among designs are made using their associated covariance matrices via the optimality criteria, including A-opt, D-opt, and E-opt. The work in [19,20] discussed the general relationship between optimality criteria and connectivity indices when using TOED for active Graph-SLAM.

Like the methods mentioned above, this paper also employed information entropy to evaluate the view and the effect of the object map. Unlike 2D/3D occupancy grid maps with grid/voxel occupancy probabilities, which can be directly used for entropy calculation, sparse object models only contain simple size and pose information. Therefore, we constructed a surface occupancy probability map and inverse sensor model for such sparse object models.

### 2.2. Object-Based Methods

Object-based view-planning methods can be divided into model-based and model-free methods. Model-free methods can be further divided into volumetric-based and surface-based methods [21]. Their application depends on how the object is modeled. Model-based methods [22,23] rely on prior knowledge of the object’s geometry or appearance. Model-free methods are more general and can adapt to the various needs of object mapping. Surface-based methods are effective when the object model is composed of curves or surfaces, such as triangle mesh modeling [24], ellipsoidal surface fitting [25], or cross-sectional B-spline fitting [26]. Under the assumption of spatial continuity, surface-based view-planning methods can predict the invisible parts of the object surface by boundary detection and surface trend analysis.

The volumetric model method is commonly used in current object mapping, i.e., octree, TSDF, and cube. Therefore, volume-based methods are more popular in view planning. In the early stages, Wong et al. [27] selected the view from which the largest number of unknown voxels of the object can be seen as the NBV. Dornhege et al. [28] extracted the boundary from the map based on the occupancy status of the voxels as the NBV. Currently, volume information gain is predominantly used. Isler et al. [16] summarized and compared five commonly used Volume Information (VI) calculation methods in object mapping, including occlusion aware VI, which considers voxel occlusion, unobserved voxel VI, which tends to explore unknown areas, rear side entropy VI, which tends to go to the back side of the object, etc. Monica et al. [29] extended frontier exploration to the TSDF model. Wu et al. [30,31] considered cube models as a whole and projected points inside the object onto surfaces, constructing a surface grid map of which the surface grids’ information entropy can represent the completeness of the object models.

Most object-based methods focus on individual objects or a singular object cluster [32], inadequately addressing complex indoor environments’ realities. To overcome this limitation, we optimized the selection of candidate views and categorized the NBVs as LBVs and GBVs. Additionally, we propose a view-planning method based on sparse object models.

## 3. View-Planning Method for Indoor Sparse Object Mapping

**Figure 1:**
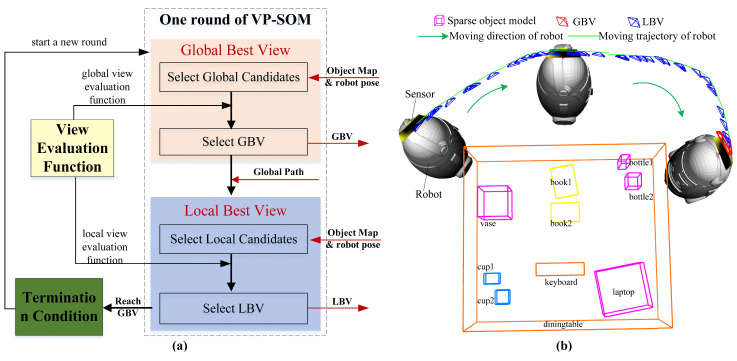
View-planning method for indoor sparse object mapping. (**a**) The workflow of VP-SOM; (**b**) a round of view planning.

The view-planning method is a core component of active mapping, with two main parts: candidate view selection and NBV evaluation. We propose a novel View-Planning method for indoor Sparse Object Mapping (VP-SOM) based on information abundance and observation continuity, as shown in Figure 1a. First, our method takes into account the properties of the object cluster. Indoor objects tend to be grouped into multiple clusters. We define an object cluster as a collection of one background object and multiple foreground objects. Background Objects (BOs) refer to large, static/semi-static, hard-to-move objects, e.g., a table, a sofa, or a cabinet. Background objects are commonly used as reliable landmarks in dynamic SLAM [33] and life-long SLAM. Foreground objects refer to small indoor objects whose positions are easily changeable, e.g., a cup, a book, or a computer. Generally, the background object provides a supporting plane for foreground objects. Objects within a cluster interact with each other during mapping, so we selected candidate views around object clusters and evaluated candidate views at the cluster level. Second, our method aims to address the unobservability problem. In addition to information abundance from candidate views, we considered observation continuity between adjacent views. This helped ensure all objects received sufficient, persistent observations throughout mapping to reconstruct accurate models.

Figure 1a shows the workflow of VP-SOM, including: the view-evaluation function, global best view, local best view, and termination condition. As shown in Figure 1b, one global best view and multiple local best views constitute a round of view planning in the active mapping. The robot moves from its starting position to the GBV. It continuously adjusts the sensor towards the LBVs using the sensor’s Degree Of Freedom (DOF) relative to the robot body. The generation process of the GBV includes the selection of global candidate views and the selection of the GBV. The goal of the GBV is to obtain more object information, to make up for objects’ information deficits and to improve the object model’s quality. Considering the characteristics of object clusters in indoor environments, the selection and evaluation of global candidate views will be processed at the level of the object cluster. The system generates multiple global candidate views around the object cluster by reading the map information and the robot’s pose. Then, the best candidate view is selected from the candidate views as the GBV using the global view-evaluation function. When there are multiple object clusters in the indoor environment, the system continues to process the same object cluster until all objects of the current object cluster are completely modeled. The GBV will be published to the robot’s motion module as the navigation goal. Unlike traditional methods that only select an NBV during one round of view planning and neglect each position during movement, our method will also constantly generate local NBVs to adjust the sensor’s posture, as the map updates and the robot moves. The generated NBV at the local position is called the local best view. The LBV-generation process includes local candidate view selection and LBV evaluation. The goal of LBVs is to ensure the continuous observation of objects, in order to optimize the quality of the object model. The local candidate views are generated uniformly within the range of activity of the sensor relative to the robot body. From these candidate views, the LBV is selected using the local view-evaluation function. The LBV will be published to the motion module to adjust the pose of the sensor. It is important to note that the view-evaluation function is critical for selecting both the GBV and LBV. Since our goal was to build a sparse object map of an indoor environment, it is essential that the view-evaluation function be constructed based on the properties of sparse object models. Specifically, we conducted in-depth research into the information abundance and observation continuity characteristics of the sparse object models. These concepts were then integrated into the design of the view-evaluation function. There are some differences between the global-view-evaluation function and local-view-evaluation function. These differences cause the GBV and LBV to have different functional focuses. When the robot reaches the determined GBV, one round of view planning ends. At this point, we check if the termination conditions have been met. If the object reconstructions are complete, view planning will terminate. Otherwise, a new round of view planning will be executed.

Algorithm 1 shows the VP-SOM. The candidate view is defined as {pose,value}, where pose is the pose of the candidate view and *value* is the evaluation value used for selecting the NBV. The NBV is defined as {pose,type}, where type is the type of NBV (GBV or LBV).

### 3.1. View-Evaluation Function

The view-evaluation function serves to select the NBV from the candidate views. Our view-evaluation function of the candidate view *v*, denoted as F(v), is *v*’s information entropy calculated from the indoor sparse object models. The function is defined as:

(1) $$F\left(v\right)=\alpha \cdot f_{a}\left(v\right)+\beta \cdot f_{c}\left(v\right)$$

The view-evaluation function F(v) consists of two parts: the object information abundance $f_{a}\left(v\right)$ and the observation continuity $f_{c}\left(v\right)$. The weights of these two parts are adjusted using α and β. The view with the maximum F(v) will be selected as the NBV from the candidate views *V*.

(2) $$NBV=\underset{v\epsilon V}{\arg\max}\left(F\left(v\right)\right)$$

$f_{a}\left(v\right)$ emphasizes obtaining more object information to quickly complete the object model, while $f_{c}\left(v\right)$ concentrates on the quality of the continuous observations of objects to minimize object mapping errors. Considering the different objectives and the real-time requirements of the GBV and LBV, we chose different α and β values in the evaluation function when selecting the GBV and LBV. We used a higher value of α (α=1.0 and β=0.2 in our experiments) in the evaluation function of the GBV, which makes the GBV focus on the object information abundance, so as to obtain information about the objects and improve the object models faster and accelerate the speed of active mapping. The value of β is higher in the evaluation function when selecting the LBV (α=0 and β=1.0 in our experiments). The LBV only depends on the observation continuity. Therefore, the LBV can be calculated in real-time based on changes in the robot’s position, reducing errors in object modeling and robot localization. See Section 3.1 for more information.

(3) $$f_{a}\left(v\right)=\sum_{fo\epsilon v}\left(H_{sopm}\left(fo\right)+H_{IoU}\left(fo\right)\right)\cdot cos\left(\theta_{sopm}\left(fo\right)\right)\cdot C_{asso}$$

where fo refers to the foreground objects within the field of view of candidate view *v*, $H_{sopm}\left(fo\right)$ is the uncertainty of fo, $H_{IoU}\left(fo\right)$ is the non-occlusion of fo, $\theta_{sopm}\left(fo\right)$ is the angle deviation between fo’s best observation LoS and the view *v*, the $C_{asso}$ is the confidence of the object–point cloud association.

(4) $$f_{c}\left(v\right)=N_{cp}\cdot \cos\left(\theta_{c}\right)$$

where $N_{cp}$ represents the co-visibility proportion between the FoV of *v* and the object cluster and $\theta_{c}$ represents the angle deviation between the object cluster’s best observation LoS and the candidate view *v*. The above components of $f_{a}\left(v\right)$ and $f_{c}\left(v\right)$ will be detailed in Section 4 and Section 5.
**Algorithm 1** VP-SOM.  **Input:** Object map *M*, robot pose *P*  **Output:** NBV  1:**while** Active mapping not end **do**  2:      Sort object clusters OC by time  3:      **for** $oc_{i}$ in OC **do**  4:            **if** $oc_{i}$ not end mapping **then**  5:                 Generate global candidates GV around $og_{i}$  6:                 **for** *v*∈GV **do**  7:                       v.value=F(v)  8:                 **for** **end for**  9:                 **for** **break**10:            **end if**11:      **end for**12:      GBV = *v* in GV with max value13:      **Publish** GBV14:      **while** P≠GBV.pose **do**15:            Generate local candidates LV in DOF of sensor16:            **for** *v*∈LV **do**17:                  v.value=F(v)18:            **end for**19:            LBV = *v* in LV with max value20:            **Publish** LBV21:      **end while**22:**end while**

### 3.2. Global Candidates and Global Best View

**Figure 2:**
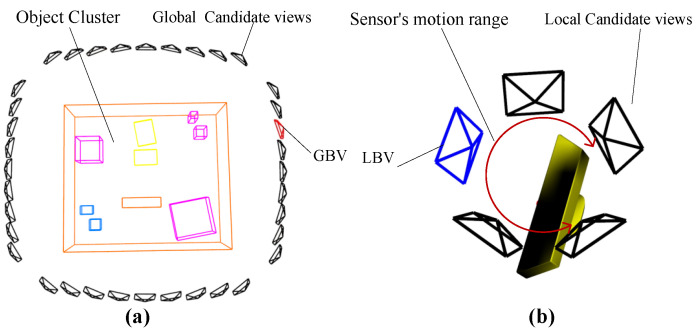
Candidate views and NBV. (**a**) Global candidate views and GBV; (**b**) local candidate views and LBV.

Lines 2–13 outline how to select global candidate views and the GBV. The GBV aims to explore unknown spaces and perceive more object information, enabling faster object mapping. Consequently, the weight of $f_{a}\left(v\right)$ in the evaluation function for the GBV is greater. As illustrated in Figure 2a, *N* candidate views are sampled uniformly around the object cluster, which is not fully modeled while maintaining a safe distance. The LoS points to the center of the object cluster. Based on (2), the GBV is selected and published as the robot exploration’s endpoint.

### 3.3. Local Candidates and Local Best View

Lines 14–21 describe how to select local candidate views and the local best view (LBV). LBVs are periodically generated when the robot moves towards the GBV. The LBV focuses on maintaining the continuous observation of the current object cluster, which helps improve mapping accuracy and reduce mapping errors. Therefore, the weight of $f_{c}\left(v\right)$ in the evaluation function for the LBV is greater. As depicted in Figure 2b, M candidate views are uniformly sampled within the sensor’s motion range. By (2), the LBV is selected and published to adjust the sensor’s pose.

### 3.4. Termination Condition

The modeling of a foreground object fo is considered complete when its uncertainty $H_{sopm}\left(fo\right)$ is less than a set threshold $\varepsilon_{sopm}$. The modeling of an object cluster OC is determined to be finished once all of its constituent foreground objects have been completed. View planning terminates when all object clusters have been completed.

## 4. Evaluation on Information Abundance

The object information abundance $f_{a}\left(v\right)$ is used to evaluate the quantity and quality of object information in the candidate view *v*’s FoV. A higher $f_{a}\left(v\right)$ indicates a view that is likely to provide more-complete and -accurate observations of objects, thereby accelerating the completion and reconstruction of object models.

### 4.1. Model Uncertainty $H_{sopm}$

**Figure 3:**
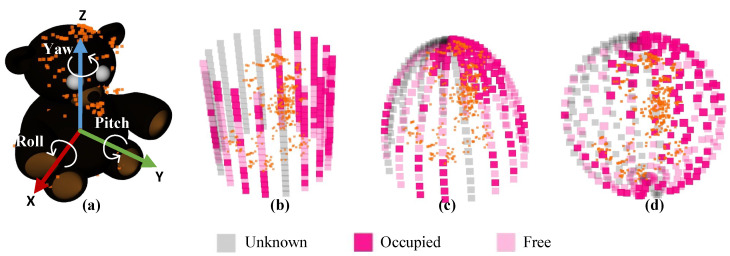
Various types of object surface occupancy probability map. (**a**) Object with 6 degrees of freedom and internal points; (**b**) cylindrical shape, suitable for camera platforms with x, y, z, and yaw DOFs, e.g., ground robot; (**c**) hemispherical shape and (**d**) spherical shape, suitable for camera platforms with full degrees of freedom, e.g., a sensor mounted on a drone.

The higher the model uncertainty $H_{sopm}$ is, the less complete the object model. To compute $H_{sopm}$ as the information entropy, we constructed a surface occupancy probability map (Figure 3), which represents the occupancy probability of each grid on the object model surface. The surface occupancy grid map revolves around the object model and is divided into (n×m) grids. The map shape can be cylindrical, spherical, hemispherical, etc., depending on the degrees of freedom of the sensor relative to the robot. The grid occupancy probabilities will be updated based on the new observations.

Based on the occupancy probabilities *p* of the grids, grid states can be classified into three states:

- Unknown: The grid is not observed, and p=0.5. This state is represented by the gray grid.
- Occupied: The grid is occupied by points, and p>0.5. This state is represented by the deep-colored grid.
- Free: The grid is observed, but not occupied, and p<0.5. This state is represented by the light-colored grid.

Initially, the occupancy probability *p* for all grids was set to 0.5, indicating an unknown state. For illustration, we uses the cylindrical surface occupancy probability map in Figure 3b to demonstrate the method for updating grid probabilities. Following [34], at time *t*, the occupancy probability $p_{t}\left(g\right)$ of grid *g* is updated in the logarithmic form $l_{t}$ as:

(5) $$l_{t}\left(g\right)={log_{2}}^{\frac{p_{t}\left(g\right)}{1-p_{t}\left(g\right)}}$$

The $l_{t}$ is updated as follows:

(6) $$l_{t}\left(g\right)=l_{t-1}\left(g\right)+l_{inv}\left(g\right)-l_{0}$$

where $l_{0}$ equals 0 because $p_{0}\left(g\right)$ equals 0.5 at Time 0. $l_{inv}\left(g\right)$ is the logarithmic form of the inverse sensor model $p_{t}\left(g|z_{t}\right)$.

(7) $$l_{inv}\left(g\right)=\log_{2}^{\frac{p_{t}\left(g|z_{t}\right)}{1-p_{t}\left(g|z_{t}\right)}}$$

**Figure 4:**
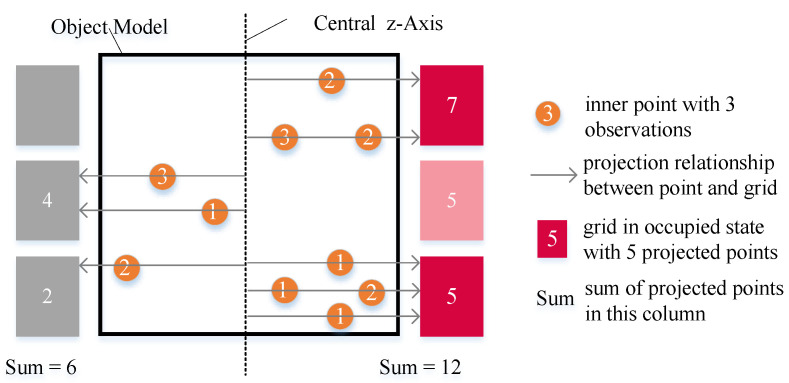
Inverse sensor model for sparse object model.

$p_{t}\left(g|z_{t}\right)$ refers to the occupancy probability of grid *g* given the sensor data $z_{t}$ at time *t*. The traditional inverse sensor model [34] does not apply to sparse object models and cannot be used to calculate $l_{inv}\left(g\right)$. Therefore, we constructed an inverse sensor model suitable for the sparse object model. We projected the inner points to the grids of the surface occupancy probability map. The projection direction varies depending on the types of object surface occupancy probability map. For the cylindrical shape, the projection direction starts from the center *z*-axis of the object model along the horizontal rays. For the spherical shape and hemispherical shape, the projection direction starts from the center of the object model along the radius. Considering the mobile robots in the experiments, we used the cylindrical object surface occupancy probability map to explain the inverse sensor model in detail, illustrated in Figure 4. Each column of the surface grids corresponds to a different observation angle. In Figure 4, inner points are projected into grids along the horizontal rays from the center *z*-axis of the object model. sum(g) is the sum of projected points in the column where grid *g* exists. $n_{p}\left(g\right)$ refers to the number of projected points in grid *g*. $n_{o}\left(g\right)$ refers to the number of observations of grid *g*. The inverse sensor model is defined as follows:

- If $sum\left(g\right)<\varepsilon_{n}$, the observation for *g* is unknown, and $p_{t}\left(g|z_{t}\right)\;=\;p_{prior}$, $n_{o}\left(g\right)=0$.
- If $sum\left(g\right)>\varepsilon_{n}$ and $n_{p}\left(g\right)>0$, the observation for *g* is occupied, and $p_{t}\left(g|z_{t}\right)\;=\;p_{occ}$, $n_{o}\left(g\right)=n_{p}\left(g\right)$.
- If $sum\left(g\right)>\varepsilon_{n}$ and $n_{p}\left(g\right)=0$, the observation for *g* is free, and $p_{t}\left(g|z_{t}\right)=p_{free}$. $n_{o}\left(g\right)$ equals the minimum observation number of occupied grids in the same column.

$\varepsilon_{n}$ is the minimum value required for valid observation and was set artificially. $p_{prior}$, $p_{occ}$, and $p_{free}$ are consistent with the traditional inverse sensor model. According to (7) and the inverse sensor model, $l_{inv}\left(g\right)$ is computed. Then, (6) is transformed into:

(8) $$l_{t}\left(g\right)=n_{o}\left(g\right)\cdot l_{inv}\left(g\right)$$

$p_{t}\left(g\right)$ is computed by (5). The information entropy H(g) of each grid *g* is defined as:

(9) $$H\left(g\right)=-p\left(g\right)\cdot log_{2}^{p(g)}-\left(1-p\left(g\right)\right)\cdot \log_{2}^{1-p(g)}$$

The model uncertainty $H_{sopm}$ of the object fo equals the average information entropy of all surface grids:

(10) $$H_{sopm}\left(fo\right)=\sum_{g\epsilon fo}H(g)/\left(N\cdot M\right)$$

### 4.2. Deviation of Foreground Object’s Best LoS $\theta_{sopm}$

**Figure 5:**
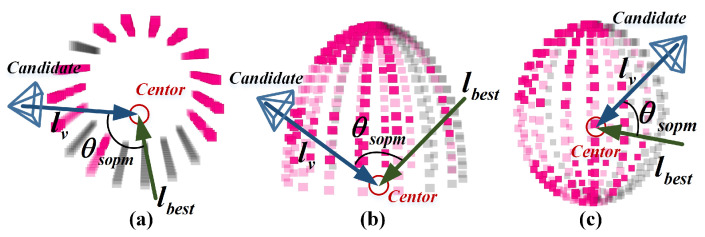
Deviation of candidate view and object’s best LoS for different types of object surface occupancy probability map. (**a**) cylindrical shape; (**b**) hemispherical shape; (**c**) spherical shape.

When the object model has only been observed from a limited range of views, grids with fewer observations tend to be unknown and to have higher uncertainty on the surface occupancy probability map. To quickly complete the object model and reduce its uncertainty, the NBV should point to unknown grid regions. We define $l_{best}$ as the best observation Line of Sight (LoS) for observing the foreground object, as shown in Figure 5.

(11) $$l_{best}\left(fo\right)=\sum_{g\epsilon fo}\left(c(g)-c(fo))\cdot H(g\right)$$

where c(g) is the coordinate of grid *g* and c(fo) is the coordinate of fo’s center. The deviation of the best LoS is defined as the angle between $l_{best}\left(fo\right)$ and the LoS $l_{v}\left(fo\right)$ of candidate view *v* pointing to the object fo.

(12) $$\theta_{sopm}\left(fo\right)=\arccos\frac{l_{best}\left(fo\right)\cdot l_{v}}{|l_{best}\left(fo\right)|\cdot |l_{v}\left(fo\right)|}$$

### 4.3. Object–Point Cloud Association Confidence $C_{asso}$

**Figure 6:**
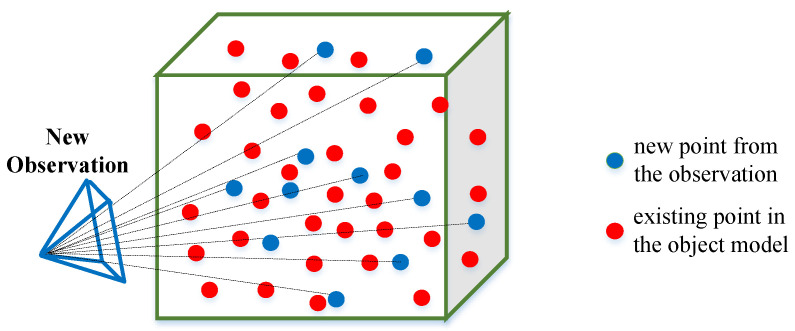
Merging between new points from observation and existing points in the object model.

The point cloud inside an object is associated and gradually merged from multiple observations and determines the quality of the sparse object model. Figure 6 shows the merging between the new object points from the new observation (blue points) and the existing or init points in the object (red points). They are judged to belong to the same object by the association method, like the semantic label, IoU, nonparametric statistic tests [7], nonparametric pose graph [35], etc. In reality, there are outliers in the point cloud and errors in the observation due to the wrong semantic recognition and wrong point extraction. Incorrect point cloud association would reduce the accuracy of the object map. Therefore, it is extremely necessary to observe objects with potential erroneous point cloud associations at close range, enrich the internal correct object point, and remove outliers. To evaluate the possibility of erroneous object point cloud associations, we designed an object–point cloud association confidence $C_{asso}$.

To verify whether points *P* from the new observation (blue points in Figure 6) and existing points *Q* in the object model (red points in Figure 6) belong to the same object, we adopted a hypothesis testing method. The null hypothesis $H_{0}$ is defined as: the point cloud *P* and the point cloud *Q* belong to the same object and have the same distribution. We calculated the test statistic to verify whether the null hypothesis is true. Considering that the point cloud distribution inside the object does not satisfy the normal distribution, we adopted the multivariate Wilcoxon rank sum test method [36]. The three-dimensional coordinates of the points in *P* and *Q* were used as the statistical data, and a multidimensional Mann–Whitney statistic U was constructed. The effectiveness of the Wilcoxon rank-sum test method for the point clouds of the sparse object model was verified in [7].

First, we merged two point clouds *P* and *Q* into one point cloud $X=\left[P|Q\right]=\left[p_{0},p_{1},\ldots ,p_{i},\ldots ,p_{\left|X\right|}\right]$, where $p_{i}$ represents a point from *P* or *Q* and |X| represents the number of points in the set *X*. We ranked the points of *X* in the three x,y,z coordinate dimensions according to the coordinate values from small to large, assigning a rank *R* to each point. We calculated the rank sums $U_{j,k}$ of *P* and *Q*, respectively, in the three x,y,z coordinate dimensions.

(13) $$U_{j,k}=\sum_{i=0}^{sum}R_{k}\left\{p_{i}\in j\right\},j=\left[P,Q\right],k=\left[x,y,z\right]$$

where $R_{k}\left\{p_{i}\in j\right\}$ represents the rank of $p_{i}$ from *j* in the *k*th dimension. We took the average approach to construct the multidimensional rank sum statistic *U*:

(14) $$U=\min\left(\frac{U_{P,x},U_{P,y},U_{P,z}}{3},\frac{U_{Q,x},U_{Q,y},U_{Q,z}}{3}\right)$$

The mean and variance of *X* is calculated as follows:

(15) $$\mu \left(X\right)=\;\frac{\left|P\right|\cdot \left|Q\right|}{2}$$

(16) $$\sigma \left(X\right)=\;\frac{\left|P\right|\cdot \left|Q\right|\cdot \left(\left|P\right|+\left|Q\right|+1\right)}{12}$$

Normalize *U* to obtain $\hat{U}$:

(17) $$\hat{U}=\frac{U-\mu (U)}{\sqrt{\sigma \left(U\right)}}$$

Calculate the probability value (*p*-value) *p*. The *p*-value *p* is a probability index used in statistical hypothesis testing to judge whether the sample observation results support or oppose the null hypothesis.

(18) $$p=\;2.0\cdot \left(1-0.5\cdot erf\left(1+\frac{\hat{U}}{12}\right)\right)\;\;$$

where erf() represents the error function, which is used to calculate the Cumulative Distribution Function (CDF) of the Gaussian distribution and the probability density of the normal distribution.

To make the null hypothesis stand, *p* should meet the following constraints:

(19) p>a

where *a* represents the significance level. We set a=0.05 in this work. In summary, if *p* is greater than the confidence level *a*, it is considered that the null hypothesis is true, and the point cloud *P* in the new observation and the object point cloud *Q* in the object model belong to the same object.

We constructed the object–point cloud association confidence $C_{asso}$ for all the object models in the map.

(20) $$C_{asso}=1-p\;\;\;\;\;$$

where *p* is the *p*-value of the point cloud association in the object’s newest observation. The closer $C_{asso}$ is to 1, the higher the possibility of errors in the point cloud fusion and the higher the priority of observing this object.

### 4.4. Non-Occlusion $H_{IoU}$

The occlusion of objects can easily lead to errors in object recognition and feature extraction. We selected the view *v* that can fully observe each foreground object according to its non-occlusion $H_{IoU}$.

To calculate the non-occlusion $H_{IoU}$ of foreground object $fo_{i}$ in the field of view of *v*, we projected $fo_{i}$ onto the image plane to obtain its 2D bounding box $b_{i}$. We calculated the IoU of bounding box between $fo_{i}$ and each other foreground object $fo_{j}$. The closer $H_{IoU}\left(fo_{i}\right)$ is to 1, the less occluded $fo_{i}$ is.

(21) $$H_{IoU}\left(fo_{i}\right)=1-\sum_{j\neq i}\frac{b_{i}\cap b_{j}}{b_{i}}$$

## 5. Evaluation on Observation Continuity

The observation continuity $f_{c}$ was used to choose the NBV, especially the LBV, which enables multi-angle continuous observation of indoor objects. This improves the quality of association between adjacent observations by achieving a more-continuous observation sequence.

### 5.1. Co-Visibility Proportion $N_{cp}$

**Figure 7:**
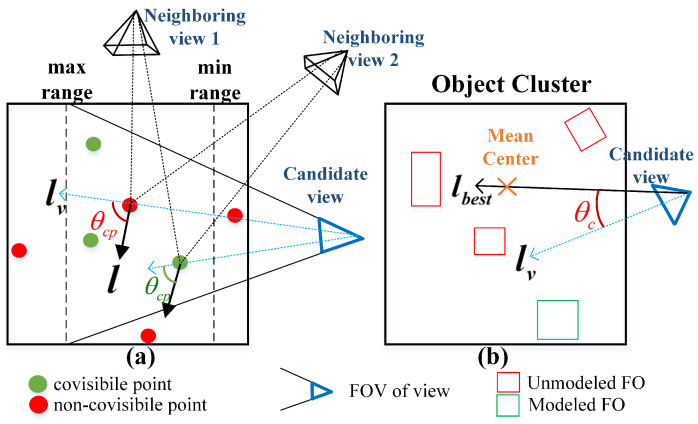
View evaluation based on the continuous observation. (**a**) Point co-visibility model; (**b**) deviation of object cluster LoS and view.

The point cloud within objects is crucial not only for selecting the NBV, but also for the quality of the object model. Thus, we propose a point co-visibility model, as shown in Figure 7a. By maximizing the number of co-visible points $n_{cp}$ between the candidate view and the object cluster tracked, we achieved better data association and improved the object mapping. The point *p* that meets the following four conditions is recorded as the co-visible point, e.g., the green point in Figure 7a:

- The point *p* is inside the FoV of candidate view *v*;
- The point *p* is inside the object cluster tracked;
- The distance between point *p* and the candidate view is within the camera’s sensing range, to ensure the performance of the point feature descriptors;
- The co-visibility angle $\theta_{cp}$ between the candidate view $l_{v}\left(p\right)$ and the old co-visible LoS l(p) is less than the threshold $\theta_{thresh}$. The old co-visible LoS l(p) is the mean LoS of point *p* in the neighboring views.

Define the co-visibility proportion $N_{cp}$ between the FoV of the candidate view and the object cluster:

(22) $$N_{cp}=\left\{\begin{array}{c} 1,if(n_{cp}\geq \varepsilon_{cp})\\ \frac{n_{cp}}{\varepsilon_{cp}},if\left(n_{cp}<\varepsilon_{cp}\right) \end{array}\right.$$

where $n_{cp}$ is the number of co-visible points and $\varepsilon_{cp}$ represents the maximum number of co-visible points, which was pre-set artificially. $N_{cp}$ was constrained within the range of [0,1] to preclude it from expanding excessively.

### 5.2. Deviation of Object Cluster’s Best LoS $\theta_{c}$

We hoped that the robot continuously observes objects that have not yet been fully modeled (the red cubes in Figure 7b). We considered the LoS from the robot to the centroid of unmodeled objects as the best observation LoS $l_{best}$, of object cluster OC. Like (12), $\theta_{c}$ is defined as the deviation between $l_{best}$ and the candidate view LoS $l_{v}$.

(23) $$\theta_{c}=\mathrm{arccocs}\frac{l_{best}\cdot l_{v}}{|l_{best}\left|\cdot \right|l_{v}|}$$

A lower $\theta_{c}$ enables more-continuous observation of the object cluster and also reduces the fluctuations in adjacent observation view angles, thereby improving the accuracy of the data association.

## 6. Active Mapping System Based on View-Planning Method for Indoor Active Sparse Object Mapping

**Figure 8:**
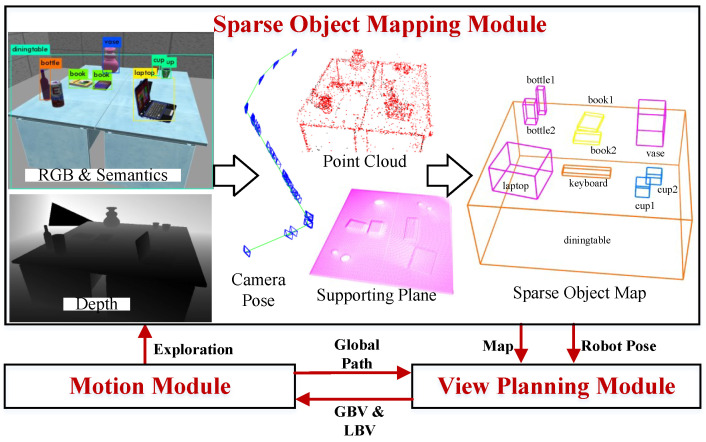
Active mapping system based on VP-SOM.

We developed an object active mapping system based on VP-SOM, illustrated in Figure 8, to validate VP-SOM. The system comprises a sparse object mapping module, a view-planning module, and a motion module. Together, these form a closed-loop for incrementally exploring and mapping indoor objects. The view-planning module selects the GBV and LBV according to the existing sparse object map and our VP-SOM method described in Section 3, then publishes them to the motion module. The motion module executes the movement of the robot chassis and sensors based on the instructions. The sparse object mapping module adopts a classic SLAM architecture for constructing a sparse object map and estimating the robot’s pose within the map. Algorithm 2 delineates the workflow of active mapping. The active mapping system cycles until no new NBV can be generated.
**Algorithm 2** Active mapping based on VP-SOM1:Initialize sparse object map *M* and robot pose *P*2:NBV = VP-SOM(*M*, *P*)3:**while** NBV not ∅ **do**4:      Move to NBV5:      Update *M*6:      NBV = VP-SOM (*M*, *P*)7:**end while**

The inputs of the sparse object mapping module consist of RGB images, depth images, and object semantic detections. To model indoor objects, we extracted point clouds and planes from the inputs and endowed them with semantic information. We then fused the point clouds belonging to the same object to form an object point cloud based on the semantic tags and the hypothesis testing method described in Section 4.3. For the foreground objects, we estimated their translation and size from their object point clouds. The point clouds within the background object tend to be highly scattered. For background objects, we approximated the space between their supporting plane and the ground as their occupied space, to estimate their translation *t* and size *s*. We calculated the objects’ orientation θ using a line alignment method [7]. The sparse object model was parameterized as $O=\left\{t,\theta ,s\right\}$. Finally, we jointly optimized the pose of the camera *C*, object *O*, and point *P* by a nonlinear optimization problem:

(24) $$\hat{C},\hat{O},\hat{P}=\underset{C,O,P}{\arg\min}\left(\sum H\left(F_{O}\right)+\sum H\left(F_{P}\right)\right)$$

where $F_{O}$ is the camera–object observation constraint and $F_{P}$ is the camera–point observation constraint. Both constraints were introduced in detail in [5,6]. Based on (24), we calculated the poses and sizes of every object and constructed a sparse object map. We also calculated the camera pose from which we can infer the robot pose.

## 7. Experiment

### 7.1. Experiment Setup

**Figure 9:**
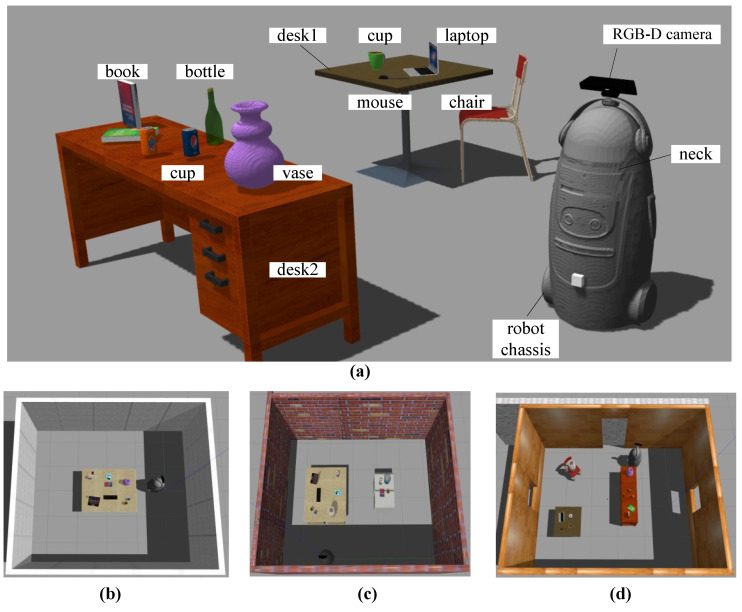
Experimental environments and robot platform. (**a**) the components of experimental environments and robot platform; (**b**–**d**) three types of simulation environments.

In this section, we conducted experiments to evaluate our VP-SOM method. The simulation environment was constructed in Gazebo and contained three indoor scenes with foreground objects (e.g., book, cup, computer) and background objects (e.g., table, chair), as shown in Figure 9a. The robot platform was the FABO humanoid robot shown in Figure 9a, equipped with a Kinectv2 RGB-D camera installed on its head. The robot can obtain RGB and depth images from the RGB-D camera and extract object semantics using YOLOv5 [37]. As FABO’s neck has a 360${}^{\circ }$ rotation range, the camera has four degrees of freedom. Since navigation was not our focus, we utilized the Robot Operating System and Cartographer’s pre-built 2D grid map. Our code and simulation environments are open-sourced at https://github.com/TINY-KE/VP-SOM (accessed on 16 November 2023).

Except for our VP-SOM method, we selected two other view-planning methods for comparison: all-views’ coverage and frontier exploration:

- VP-SOM: We applied Algorithm 1 to generate the GBVs and LBVs and directed the robot to autonomously explore the indoor environment. The robot navigated to the GBVs using its chassis navigation. Simultaneously, the robot continuously rotated its neck to align the camera with the LBVs. Foreground object mapping ended when the model uncertainty was less than 0.42.
- All-views’ coverage: The robot walked a complete circle around every object cluster, while its top camera constantly pointed at the center of the current object cluster, ensuring coverage of all observation angles of the object cluster. Every view on the observation trajectory of this method can be considered as one LBV. This method ended when the robot revolved around each object cluster once.
- Frontier exploration: According to [10], frontiers of 3D point cloud were used as GBVs to guide robot exploration of the indoor environment. This method ended when there are no more reachable frontiers in the map.

**Figure 10:**
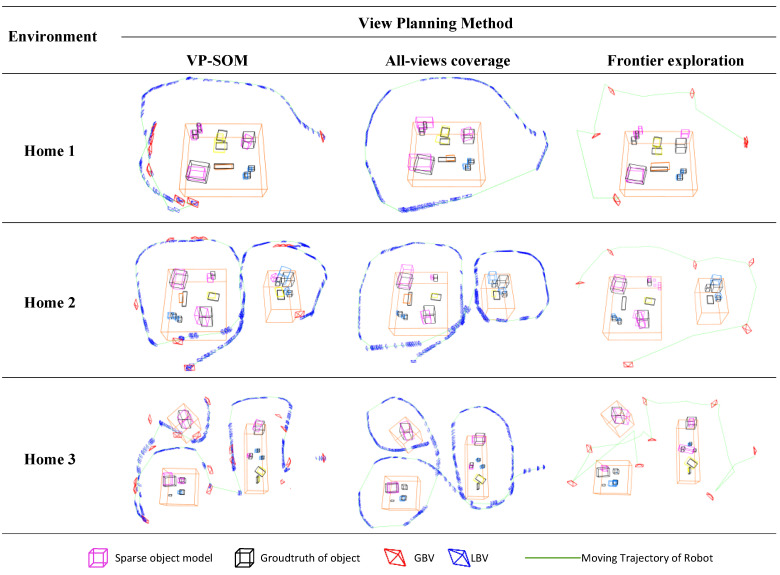
Results of sparse object map and observation trajectory.

We conducted five experiments for each of the three methods in every simulation environment in Figure 9b–d. Figure 10 depicts the results of the sparse object maps and observation trajectories generated by the three methods in the parts of the experiments. In Figure 10, each colored cube represents an object, with its geometry indicating the object’s pose and size and its color indicating its semantic type. The black cubes denote the ground-truth objects extracted from the simulation environment. The ground-truth objects can be used to evaluate the accuracy and precision of the sparse object maps. The blue pyramids represent the local best view of each method, while the red pyramids represent the GBV.

The following will discuss the three types of methods in terms of the sparse object map and observation trajectory.

### 7.2. Sparse Object Map

The objective of object active mapping is to autonomously build an object map without human intervention. Therefore, we evaluated each method based on the accuracy and precision of the generated object maps. The evaluation metrics were as follows:

- Precision: An object was considered to be accurately modeled if its semantic label matched its ground-truth and the center distance was less than 0.1 m. Precision is the ratio of the number of correct models $n_{succ}$ to the total number of models $n_{model}$.
- Recall: Recall is the ratio of the number of correct models $n_{succ}$ to the total number of ground-truths $n_{gt}$.
- IoU: Align the centers and orientations of the object model and its ground-truth, then calculate their 3D IoU, which reflects the size accuracy of the object model.
- Center Distance Error (CDE): the center distance (in meters) between the object model and its ground-truth.

Table 1 displays the evaluation results of the sparse object maps by the three methods. Our method significantly improved the accuracy and precision of the object maps compared to the other methods.

Our method took into account indoor characteristics and object information abundance, which ensured that most objects received sufficient observation. The frontier exploration method ignored objects, resulting in poor and insufficient object observations. Its accuracy and recall were the lowest (50.0% and 29.9% lower than ours). Although all-views’ coverage guaranteed the complete observation of the indoor objects, it did not consider the quality of information, so its accuracy and recall were 30.1% and 9.3% lower than ours. Our method acquired less-erroneous information through non-occlusion and more-accurate information by increasing the observations of complex regions and objects (such as the bottles and notebook in Figure 10 of Home 1).

In addition to higher-quality data, our method improved the data association through observation continuity, making object poses and sizes more accurate. Our 3D IoU was 33.1% and 36.8% higher than all-views’ coverage and frontier exploration, respectively, and the CDE was 32.9% and 75.7% lower than them.

### 7.3. Observation Trajectory of Active Mapping

On the basis of ensuring the accuracy and precision of the object map, the observation process of object active mapping should be efficient and robust. We evaluated the observation trajectories of each method based on the following metrics:

- Trajectory length: The distance traveled by the robot’s chassis from the start to the end of active mapping.
- Object non-occlusion degree: Calculate the average non-occlusion degree of the objects from all NBV perspectives according to (21).
- Number of localization failures: When visual localization failed in SLAM, we let the robot keep moving until successful relocalization. If the time interval between failure to localize and successful relocalization exceeded 1 s and the distance exceeded 0.3 m, the number of failures increased by one.

Table 2 displays the evaluation results of the observation trajectories generated by the three methods in the three experimental scenarios. In terms of the trajectory length, while frontier exploration was the shortest, it did not focus on observing objects, leading to terrible object mapping results. Compared to all-views’ coverage, our method reduced the trajectory length by 17.1% while ensuring map quality.

The object non-occlusion degree of our method was 56.5%, which was 27.7% higher than all-views’ coverage, which is one of the reasons why our method reduced the wrong observations and improved the data quality. Our average number of localization failures was 20.6% and 55.8% lower than the other two methods, because our continuous, robust, and accurate observations ensured SLAM safety. When the robot lost its localization, it needed to rotate or draw back to relocalize, which cost much time. Therefore, our method’s cost time was less than the other two by 28.34% and 16.44%, respectively, despite our trajectory not being the shortest.

As is evident from Figure 10, the active mapping’s observation trajectory generated by our method prioritized objects that were challenging to model and viewed with less object occlusion. Hence, the robot did not have to fully circle the object clusters, thereby saving exploration time and distance. Simultaneously, the high-quality observation information enhanced the robustness and safety of localization.

### 7.4. The Role of Each View-Evaluation Item

To better understand the role of each term in the view-evaluation function (1), we demonstrate and analyze the intermediate dataof each view-evaluation item when choosing the GBV and LBV in Figure 2.

**Figure 11:**
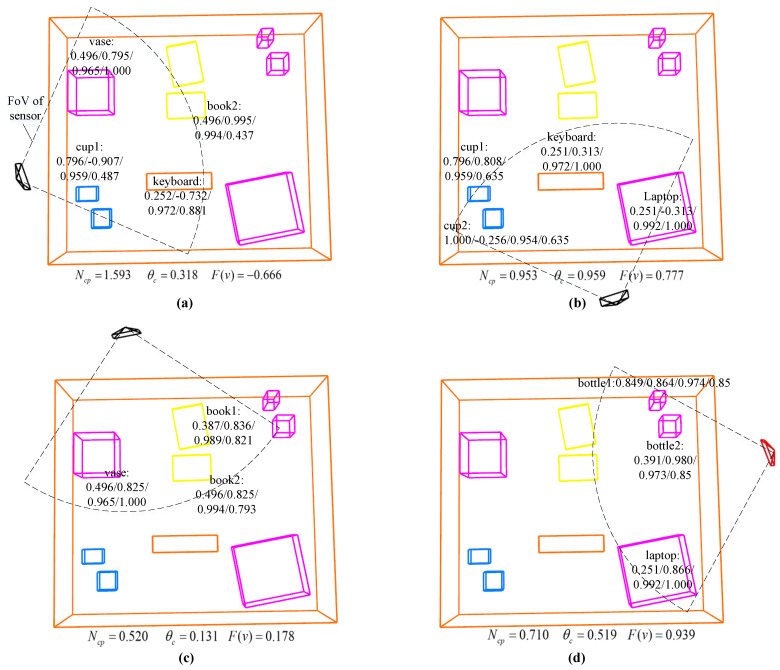
Intermediate data during the global candidate view evaluation. (**a**–**d**) demonstrate the evaluation process of four global candidate views. The object’s model uncertainty $H_{sopm}$, deviation of the best LoS $\theta_{sopm}$, object–point cloud association confidence $C_{asso}$, and non-occlusion $H_{IoU}$ are shown around the object model. The co-visibility proportion $N_{cp}$, the deviation of the object cluster’s best LoS $\theta_{c}$, and the final evaluation value F(v) are displayed below the each image. Some objects with significant modeling deviations are not displayed in the object map. The red view in (**d**) with largest evaluation value is GBV.

When choosing the GBV, VP-SOM selected 36 candidate views (Figure 11 shows parts of the candidates) around the background objects and calculated the model uncertainty $H_{sopm}$, the deviation of the best LoS $\theta_{sopm}$, the object–point cloud association confidence $C_{asso}$, and the non-occlusion $H_{IoU}$ for each object (shown around the object model) within the field of view of the candidate views. We also calculated the co-visibility proportion $N_{cp}$ and the deviation of the object cluster’s best LoS $\theta_{c}$ for the current background object. According to Equation (1), the evaluation value F(v) of each candidate view *v* was calculated. The candidate view with the highest evaluation value was selected as the GBV. In Equation (1), we applied α=1.0 and β=0.2, such that the GBV tended to focus on the information richness, in order to acquire the object information and complete the object models faster. At this stage of active mapping, most objects were basically completed, except Bottle 1, Cup 1, and Cup 2 with uncertainties of 0.849, 0.796, and 1.000, respectively. Therefore, candidate views that can observe and supplement these three objects with the best observation angle and minimum occlusion received a higher information abundance evaluation value. Considering that the deviation of the best LoS for Cup 2 of the candidate in Figure 11b was bad, the system chose the candidate in Figure 11d as the GBV, which had a good observation view for Bottle 1. Nonparametric tests were used to merge the internal point clouds of the objects during the object modeling, and the significance level was set to 0.05, so the object–point cloud association confidence $C_{asso}$ was above 0.95, with little impact on the evaluation value F(v).

When choosing the LBV, the system selected 18 candidate views (Table 3 only shows a part of the candidates) within the range of activity of the sensor relative to the robot body and calculated the co-visibility proportion $N_{cp}$ and the deviation of the object cluster’s best LoS $\theta_{c}$ for the current background object. The evaluation value of each candidate view F(v) was calculated. The candidate view with the highest evaluation value was selected as the LBV. For the LBV selection, we applied α=0 and β=1.0 in Equation (1) such that the LBV depended entirely on the observation continuity. Robot localization relies on object SLAM, and the continuous observation of the object can improve the robustness of object SLAM and reduce the number of localization failures. Moreover, the LBV needs to be calculated in real-time by the changes of the robot’s position, while the computation of the information abundance was somewhat slow and did not meet real-time requirements during fast robot movement.

## 8. Conclusions

In summary, we proposed a view-planning method for indoor sparse object mapping based on information abundance and observation continuity during active mapping. This approach is well suited for coexisting human–robot environments by taking into account for the first time the properties of object clusters. Our view-planning method incorporates a view-evaluation function, a global best view selection, a local best view selection, and a termination condition. In particular, we constructed an object surface occupancy probability map and a point co-visibility model for sparse object models to incorporate them into the view-evaluation function. Multiple experiments in indoor environments were conducted to verify our method. By the comparison of the object maps and observation trajectories, the experimental results showed that our method guided the indoor object active mapping more efficiently and accurately.

For future work, we plan to expand the mapping scenario to multiple interconnected rooms and focus on improving the efficiency of multi-room exploration. We will apply our view-planning method to other robotic platforms like robotic arms and drones, integrating it with motion planning to enhance the overall performance of active mapping. We also intend to continue our in-depth research on information abundance and observation continuity to adapt the approach to more-complex object models, such as those represented by DeepSDF [8]. This will allow us to map environments with a wider variety of object shapes.

## Figures and Tables

**Table 1:** Comparison of sparse object map. VP-SOM, Cover, Frontier denote proposed VP-SOM, All-views’ coverage, Frontier exploration, respectively. Scene 1–3 corresponds to Figure 9b–d, respectively. Bold numbers represent the best performances.

Scene	Metrics	VP-SOM	Cover	Frontier
1	Precision	**0.90**	0.50	0.38
	Recall	**1**	1	0.67
	IoU	**0.769**	0.558	0.590
	CDE(m)	**0.041**	0.084	0.113
2	Precision	**0.79**	0.64	0.41
	Recall	**1**	0.82	0.63
	IoU	**0.789**	0.562	0.728
	CDE(m)	**0.089**	0.127	0.103
3	Precision	**0.59**	0.45	0.35
	Recall	**0.91**	0.82	0.73
	IoU	**0.795**	0.647	0.401
	CDE(m)	**0.052**	0.062	0.534
Ave	Precision	**0.76**	0.53	0.38
	Recall	**0.97**	0.88	0.68
	IoU	**0.784**	0.589	0.573
	CDE(m)	**0.061**	0.091	0.250

**Table 2:** Comparison of observation trajectory. VP-SOM, Cover, Frontier denote proposed VP-SOM, All-views’ coverage, Frontier exploration, respectively. Scene 1–3 corresponds to Figure 9b–d, respectively. Bold numbers represent the best performances.

Scene	Metrics	VP-SOM	Cover	Frontier
1	Length of path	10.39	12.05	**8.58**
	Cost time	**248**	295	273
	Non-occlusion	**0.631**	0.426	0.279
	Localization failure	**3**	4	7
2	Length of path	17.94	21.22	**11.25**
	Cost time	**385**	463	472
	Non-occlusion	**0.601**	0.519	0.200
	Localization failure	**7**	9	14
3	Length of path	18.80	23.62	**19.55**
	Cost time	**664**	830	807
	Non-occlusion	**0.464**	0.383	0.239
	Localization failure	**5**	6	13
Ave	Length of path	15.71	18.96	**13.13**
	Cost time	**432**	529	517
	Non-occlusion	**0.565**	0.443	0.239
	Localization failure	**5**	6	11

**Table 3:** Intermediate data during the local candidate view evaluation. Local candidate 1–7 are parts of the local candidate views. Evaluation value is the evaluation result computed by Equation 1. Bold number represent the largest value. The candidate view corresponding to the bold number is LBV.

Local Candidate	1	2	3	4	5	6	7
$N_{cp}$	0	0.073	0.637	0.9	1.383	0.773	0.133
$\theta_{c}$	−0.494	0.111	0.413	0.674	0.979	0.994	0.739
**Evaluation value**	0	0.008	0.263	0.607	**1.354**	0.768	0.098

## Data Availability

Data are contained within the article.
